# Moving forward: embracing challenges as opportunities to improve medical education in the post-COVID era

**DOI:** 10.1057/s41599-022-01451-7

**Published:** 2022-11-26

**Authors:** Kyong-Jee Kim

**Affiliations:** Department of Medical Education, Dongguk University School of Medicine, Goyang, South Korea

**Keywords:** Education, Science, technology and society

## Abstract

Pandemics affect every aspect of life, and Coronavirus Disease 2019 (COVID-19) is no exception. The impact of COVID-19 might be even greater in medical education, which involves close contact with patients. This comment reviews current trends in medical education in response to COVID-19, especially in the pre-clerkship curriculum, and discusses opportunities and challenges in medical education in the post-pandemic era. COVID-19 has accelerated the adoption of online teaching and learning and is expected to boost innovation in medical education. First, blended learning, which is a mix of online and offline learning intended to incorporate the best of both worlds, is expected to become more widespread. Second, more novel approaches to learning that involve student-led initiatives likely become popular mediated by various technologies. Third, there will be more use of online learning resources and assessments. As online learning is expected to play a prominent role in the post-COVID-19 era, such transitions offer both opportunities and challenges. These challenges include faculty development on online teaching skills, creation and sharing of online resources, and effective design and implementation of online assessments. This comment calls for institutional support and collaborations for faculty development and for the development and sharing of learning resources, more models and guidelines for effective technology integration, and use of the virtual learning environment to promote student-centered learning to embrace the challenges as opportunities to improve medical education in the post-COVID era.

## Introduction

Pandemics have worldwide repercussions, as has been amply demonstrated by Coronavirus Disease 2019 (COVID-19), and their effects on medical education are substantial because it requires close contact with patients. Historically, this was also experienced during SARS, which was prevalent in 2003. To prevent class interruptions caused by SARS outbreaks, online Problem-Based Learning (PBL) was introduced at a medical school in China and was subsequently continued because of excellent feedback from professors and students (Ahmed, Allaf, and Elghazaly 2020). Similarly, in Hong Kong, SARS prompted the use of technology in PBL in medical education (Patil, Chan, and Yan 2003). Moreover, the COVID-19 pandemic constitutes an ongoing threat, and thus, is promoting continuous innovation rather than temporary changes in medical education (Gill, Whitehead, and Wondimagegn 2020; Rose 2020).

Given the experience of online teaching and learning caused by COVID-19, it is believed that the changes adopted in medical education will be further promoted. The futurist Jason Schenker (2020) predicted that online education for healthcare professionals would emerge as one of the most promising fields in the post-COVID era. As such, changes triggered by COVID-19 impose both challenges and opportunities for medical education and provide an opportunity to rethink our overall approach to medical education (Dent, Harden, and Hunt 2021). Although several articles have described how medical schools have responded to the pandemic (Daniel et al. 2021; Gill et al. 2020; Gordon et al. 2020; Woolliscroft 2020), discussions regarding its impact on the future trends of medical education are scant. This comment discusses the impacts of the pandemic on medical education and its prospects in the post-COVID era and argues how to embrace the challenges as opportunities to improve medical education, particularly in the pre-clerkship phase of basic medical education.

## Impacts of the pandemic on medical education and its prospects

### Blended learning: the new normal for teaching and learning

As a result of the rapid transition to online learning in response to the pandemic, we have experienced a variety of learning environments that transcend traditional classroom instruction. e-Learning has become a mainstream educational method since it was introduced in the early 2000s (Ellaway and Masters 2008). Even if not all students are taught remotely after COVID-19, it is likely that the use of blended learning, that is, a mix of traditional classroom instruction and online instruction, will become a popular mode of instruction in medical education. Blended learning aims to enhance the effectiveness of education by utilizing the advantages of face-to-face and online learning. This form of teaching and learning has been used in higher education for around 20 years, and research on blended learning in health professions education have shown it is more effective than traditional classroom instruction in terms of knowledge acquisition (Liu et al. 2016; Vallée, Blacher, Cariou, and Sorbets 2020). Still, there is scant research that informs the effective practice of blended learning in medical education. Thus, more research is warranted to provide theoretical and practical backgrounds on effective blended learning models.

### More student-centered learning environments

Abrupt changes in medical education curricula during the pandemic resulted in efforts to incorporate more student-led learning activities. One of the benefits of online learning is that it facilitates collaboration between institutions, which reduces duplication and benefits large numbers of learners (Chen et al. 2019). A recent study showed that student suggestions and translations of educational resources on the COVID-19 pandemic have had positive influences on student learning and reached wider audiences (Kochis and Goessling 2021). In another study, medical students participated in service learning which involved student participation in initiatives that contributed to healthcare in their local communities, such as collecting and distributing personal protective equipment and providing childcare for frontline workers (Nguemeni Tiako, Johnson, Nkinsi, and Landry 2021). Those cases illustrate that there have been efforts to sustain student-led learning activities even during the time of the pandemic. More novel approaches to learning that involves student-led initiatives likely become more popular enabled by the use of various technologies. Such experiences will pave the way for more innovative and noble programs in which medical students reach out and collaborate nationally and internationally.

In particular, online learning is expected to promote more student-centered learning by offering customized learning for individual learners that enables learning on demand and at his or her own pace, increasing social interactions mediated by technology, and providing convenient access to learning resources (Han et al. 2019). Moreover, the virtual learning environment rapidly adopted during the COVID-19 pandemic will likely bring about increased student autonomy in both small-group and large-group learning settings (Belovich et al. 2022). Increased student autonomy can lead to positive learning outcomes, such as a higher student engagement, increased intrinsic motivation and responsibility for learning. Such a learning environment is conducive to fostering lifelong learning attitudes in students (Belovich et al. 2022).

### Challenges for enhancement of the quality of online education

Technology use introduces many risks and challenges to medical education as well as many advantages and opportunities (Ellaway 2019). Among such challenges are lack of faculty competence, learning resources, and institutional strategies and support (O’Doherty et al. 2018). As online learning is expected to play a prominent role in the post-COVID era, several challenges need to be addressed to improve the quality of online teaching and learning in medical education.

#### Faculty development in online education

Although online learning has become prevalent in medical education, simply converting traditional teaching methods to an online format cannot lead to effective student learning. Teachers with no experience of online teaching tend to adopt traditional teaching methods, which are known as ineffective methods such as lectures, to online teaching (Baran, Correia, and Thompson 2011). In particular, various roles are emphasized by instructors to teach online, not only in delivering the content, but also in planning learning activities and providing social support to learners (Baran et al. 2011). In addition, many technologies can be used to promote learner collaboration and creative activities in the online learning environment, and thus, teachers should be familiarized with these technologies. Therefore, a support system is required at the university level that provides individual support to instructors with questions or requests for help about online teaching.

#### Models and guidelines for effective technology integration

Online learning can be broadly divided into synchronous and asynchronous modes depending on the manner in which interactions take place amongst participants, such as between teachers and learners (Rhim and Han 2020), which have different strengths and characteristics (see Table 1). During synchronous online learning, participants interact in real-time. Recently, developments of various distance learning platforms have popularized synchronous online learning, which has the advantage of enabling interactions and providing instant feedback. On the other hand, during asynchronous online learning, interactions between teachers and students occur later during lecture videos and discussion forums, which allow students time to think about topics beforehand. Asynchronous learning has the advantage of facilitating learning using group interactions between participants or participants and distant groups (Serdyukov 2020).

**Table 1:** Comparisons of the two types of online learning.

Synchronous learning	Asynchronous learning
Real time, live interaction	Delayed (or lack of) live interaction
Social learning	Independent learning
Immediate feedback	Delayed response, limited feedback
Reactive, alert	Self-paced, deeper learning
Limitation on flexibility	Flexible, time efficient
Organized and facilitated by the instructor	Scarce guidance and support
May have to deal with diverse situations	High time management expectations, risk of procrastination
Real-time discourse, debate, discussion	More time on task and reflection

^*^Adapted from Serdyukov P. Asynchronous/synchronous learning chasm. In: Mahoney J, Hall CA, editors. Exploring online learning through synchronous and asynchronous instructional methods. Hershey, Pennsylvania: IGI Global; 2020. p. 1-33.

Given the advantages and disadvantages of the two different types of online learning, a holistic approach is warranted for the effective design and implementation of online classes and follow-up activities (Serdyukov 2020; Stojan et al. 2021). Although the potential of technology use to transform teaching and learning is well known (Ellaway 2019), a recent systemic review of online learning in medical education shows that it was largely used to replace or amplify traditional teaching for online classes, and its potential to transform teaching remains largely unrealized (Stojan et al. 2021). More research is warranted to identify the theoretical and practical implications of effective technology integration in medical education, especially on when and how to use two different types of online learning.

#### Creation and sharing of learning resources, and online assessments

Adequate resources for supporting student learning are of paramount importance in the online learning environment. Learning resources can be utilized in diverse ways depending on the teaching methods used and educational situations, such as those of team-based and flipped learning, which promote active learning. For example, the Massive Open Online Course (MOOC) is attracting attention as an important learning method for online learning, and learning resources incorporated in the MOOC, such as lecture videos, online reading materials, and quizzes can also be used for classroom teaching (de Jong et al. 2020). Still, it takes a significant amount of medical faculty time and effort to develop such resources, which is more challenging for those in resource-constrained settings. Thus, more innovative, efficient ways of developing and sharing them are warranted through collaborations among medical schools.

Learning resources may include assessment items, which can be used to provide feedback or assess the academic achievements of learners. Online assessments have the advantage of mediating technology to provide more robust assessments, which is emphasized in the concept of ‘technology-enhanced assessment’ (Dennick, Wilkinson, and Purcell 2009; Ellaway 2019; Fuller et al. 2022). Still, using technology in assessments may be vulnerable to student misconduct, especially in high-stakes assessments. To remedy this issue, alternatives such as the use of assessment methods with a low probability of student cheating (essays, open-book tests, etc.) or secure technologies could be used (Dennick et al. 2009). However, since there may be technical limitations in preventing student cheating in online exams, there is also an opinion that emphasizes the need for professionalism education to keep students academically honest in student assessment (Association for Medical Education in Europ (2020)). Moreover, adoption of technology in assessment, by itself, does not ascertain enhancement of the quality of assessment; rather, it stills depends on employing robust general assessment principles (Ellaway 2019). Therefore, medical teachers need to familiarize themselves with various online assessment tools that are available to them and how to use them effectively as well as to make continuous efforts to improve their skills for robust general assessment principles.

## Conclusions: moving forward

The COVID-19 pandemic is expected to bring about several changes in medical education. Future trends in medical education include blended learning, more student-centered learning and curricula mediated by various technologies, and more use of online learning resources and assessments. The digital transformation is expected to offer medical students with the opportunities for technology-enhanced learning and the learning environment that promotes lifelong learning (Belovich et al. 2022, Thibault 2020). However, there are several challenges for this digital transformation, which include faculty development on online teaching skills, creation and sharing of online resources, and effective design and implementation of online assessments.

This comment has implications for moving forward to embrace the challenges as opportunities to improve medical education, particularly in the pre-clerkship phase of basic medical education. First, faculty development is warranted for medical faculty to help them acquire the competencies for the changing and various roles of teachers in the technology-mediated learning environment. Second, more models and guidelines for effective technology integration are needed to improve the current practice of online teaching and learning in medical education that moves beyond replacing or amplifying traditional teaching. Third, collaborations are suggested for medical schools to develop and share learning resources and to provide medical students with more innovative and noble learning environments in which medical students reach out and collaborate in a wide variety of settings.
